# CHFR and Paclitaxel Sensitivity of Ovarian Cancer

**DOI:** 10.3390/cancers13236043

**Published:** 2021-11-30

**Authors:** Andrea E. Wahner Hendrickson, Daniel W. Visscher, Xiaonan Hou, Krista M. Goergen, Hunter J. Atkinson, Thomas G. Beito, Vivian Negron, Wilma L. Lingle, Amy K. Bruzek, Rachel M. Hurley, Jill M. Wagner, Karen S. Flatten, Kevin L. Peterson, Paula A. Schneider, Melissa C. Larson, Matthew J. Maurer, Kimberly R. Kalli, Ann L. Oberg, S. John Weroha, Scott H. Kaufmann

**Affiliations:** 1 Division of Medical Oncology, Mayo Clinic, Rochester, MN 55905, USA; hou.xiaonan@mayo.edu (X.H.); wagner.jill@mayo.edu (J.M.W.); Weroha.Saravut@mayo.edu (S.J.W.); 2 Department of Laboratory Medicine and Pathology, Mayo Clinic, Rochester, MN 55905, USA; Visscher.Daniel@mayo.edu; 3 Department of Quantitative Health Sciences, Mayo Clinic, Rochester, MN 55905, USA; Goergen.Krista@mayo.edu (K.M.G.); Atkinson.Hunter@mayo.edu (H.J.A.); Larson.melissa@mayo.edu (M.C.L.); Maurer.Matthew@mayo.edu (M.J.M.); oberg.ann@mayo.edu (A.L.O.); 4 Hybridoma Core, Mayo Clinic, Rochester, MN 55905, USA; Beito.Thomas@Mayo.edu; 5 Pathology Research Core, Mayo Clinic, Rochester, MN 55905, USA; Negron.Vivian@mayo.edu (V.N.); wilmalingle@yahoo.com (W.L.L.); amybruzek@gmail.com (A.K.B.); 6 Department of Molecular Pharmacology & Experimental Therapeutics, Mayo Clinic, Rochester, MN 55905, USA; rachelhurley.mayo@gmail.com; 7 Division of Oncology Research, Mayo Clinic, Rochester, MN 55905, USA; flatten.karen@mayo.edu (K.S.F.); Peterson.kevin1@mayo.edu (K.L.P.); Schneider.paula@mayo.edu (P.A.S.); 8 Women’s Cancer Program, Mayo Clinic, Rochester, MN 55905, USA; rossman.kristi@mayo.edu

**Keywords:** CHFR, ovarian cancer, taxanes, patient-derived xenografts

## Abstract

**Simple Summary:**

Studies in tissue culture cell lines have indicated that silencing of the mitotic regulator CHFR is associated with increased paclitaxel sensitivity. More recently, it has been suggested that a UBC13-DNMT1-CHFR pathway also modulates sensitivity of ovarian cancer to paclitaxel in the clinic. Here we have credentialed an anti-CHFR monoclonal antibody for immunohistochemistry and directly examined the association of CHFR expression with outcome of paclitaxel-containing ovarian cancer therapy. While CHFR levels were higher in high grade, high stage ovarian cancer, after correction for stage and debulking status there was no association between CHFR levels and progression-free survival in high grade serous ovarian cancer treated with surgery followed by platinum/taxane therapy. Moreover, there was no association between CHFR expression and response of patient-derived ovarian cancer xenografts treated with paclitaxel monotherapy. These studies indicate that CHFR varies among ovarian cancers but is unlikely to be an independent biomarker for poor response to taxanes.

**Abstract:**

The poly(ADP-ribose) binding protein CHFR regulates cellular responses to mitotic stress. The deubiquitinase UBC13, which regulates CHFR levels, has been associated with better overall survival in paclitaxel-treated ovarian cancer. Despite the extensive use of taxanes in the treatment of ovarian cancer, little is known about expression of CHFR itself in this disease. In the present study, tissue microarrays containing ovarian carcinoma samples from 417 women who underwent initial surgical debulking were stained with anti-CHFR antibody and scored in a blinded fashion. CHFR levels, expressed as a modified H-score, were examined for association with histology, grade, time to progression (TTP) and overall survival (OS). In addition, patient-derived xenografts from 69 ovarian carcinoma patients were examined for CHFR expression and sensitivity to paclitaxel monotherapy. In clinical ovarian cancer specimens, CHFR expression was positively associated with serous histology (*p* = 0.0048), higher grade (*p* = 0.000014) and higher stage (*p* = 0.016). After correction for stage and debulking, there was no significant association between CHFR staining and overall survival (*p* = 0.62) or time to progression (*p* = 0.91) in patients with high grade serous cancers treated with platinum/taxane chemotherapy (N = 249). Likewise, no association between CHFR expression and paclitaxel sensitivity was observed in ovarian cancer PDXs treated with paclitaxel monotherapy. Accordingly, differences in CHFR expression are unlikely to play a major role in paclitaxel sensitivity of high grade serous ovarian cancer.

## 1. Introduction

The Checkpoint with Forkhead-associated and RING finger domains (CHFR) protein is a cell cycle checkpoint component that, based on its frequent mutation [1]or methylation-induced silencing [2,3], is thought to be a tumor suppressor [4,5]. In addition to binding poly(ADP-ribose) [6,7,8], CHFR serves as an E3 ubiquitin ligase to regulate levels of the mitotic kinases Polo-like Kinase 1 [9] and Aurora A [10] as well as several other proteins [11,12,13]. By downregulating Polo-like kinase 1 (PLK1) and/or Aurora A, CHFR is thought to diminish cyclin dependent kinase 1 activity and delay entry into mitosis.

Studies in isogenic cell lines have established a role for CHFR in sensitivity to spindle poisons. In particular, when microtubule dynamics are disrupted by agents such as paclitaxel [14,15], cells containing CHFR arrest in G2 prior to entering mitosis [16,17] or even back up from early mitosis into late G2 [18], where they are somewhat protected from cell death. In contrast, CHFR deficient cells fail to arrest in late G2 in response to these agents [16,19] and instead enter mitosis, arrest because of spindle assembly checkpoint activation, and acquire lethal damage that leads to apoptosis upon checkpoint adaptation [16,19,20]. Accordingly, cells with CHFR loss are more sensitive to spindle poisons such as paclitaxel [16,21].

The *CHFR* gene is frequently inactivated in cancer cells [1,2,3,4,5]. CpG island methylation within the *CHFR* promoter is associated with a poor prognosis in multiple cancer types [5], including microsatellite stable colorectal cancer [22,23]. On the other hand, *CHFR* promoter methylation is also associated with increased taxane sensitivity in colorectal cancer [24], gastric cancer [25], and nonsmall cell lung cancer [26]. Likewise, silencing of *CHFR* is associated with increased taxane sensitivity in endometrial [27,28] and cervical cancer cell lines [29].

Ovarian carcinomas are the most lethal of all gynecologic malignancies [30]. Current first line treatment includes debulking surgery and adjuvant or neoadjuvant therapy with a combination of a platinum and a taxane, usually paclitaxel [31]. Taxanes are also used in the recurrent setting for both platinum sensitive and resistant ovarian cancers [32]. Despite this widespread use of taxanes, little is known about CHFR in ovarian cancer. One early study indicated that the *CHFR* gene is neither methylated nor mutated in ovarian cancer [33], whereas a subsequent report indicated that the *CHFR* gene is hypermethylated and downregulated at the mRNA level [34]. More recently, the deubiquitinase UBC13, acting through its substrate DNMT1, was shown to cause increased methylation of the *CHFR* locus and diminished CHFR expression [35]. Importantly, UBC13 was also shown to be lower in ovarian cancer samples that had a shorter disease-free survival after paclitaxel-containing therapy, leading to the conclusion that low UBC13 and high CHFR might be mediators of paclitaxel resistance in ovarian cancer [35]. Because of the substantial toxicities of paclitaxel, including peripheral neuropathy and leukopenia [36], the ability to identify patients most likely to respond would be beneficial clinically.

None of these studies directly examined the CHFR protein or assessed the relationship between CHFR expression and treatment outcome. In the present study, we describe the generation and credentialing of an anti-CHFR antibody specifically generated for immunohistochemistry (IHC), then use this antibody to examine the relationship between CHFR expression and treatment outcome in newly diagnosed epithelial ovarian cancers treated with adjuvant platinum/taxane therapy and in ovarian cancer patient-derived xenografts (PDXs) treated with paclitaxel monotherapy.

## 2. Results

### 2.1. Development of an Antibody for Studying CHFR Expression

**Figure 1:**
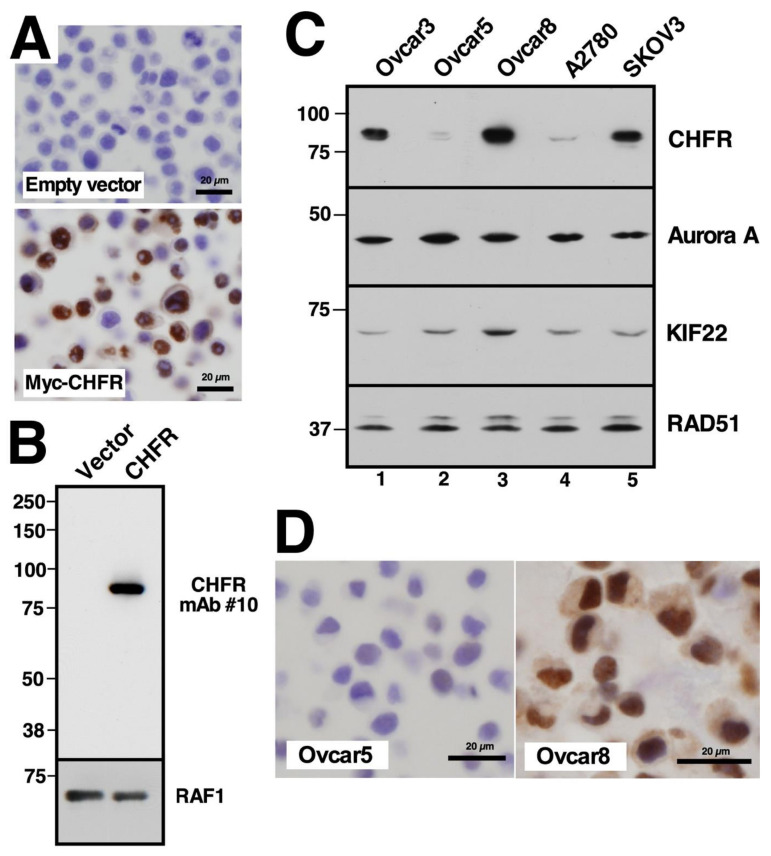
CHFR antibody characterization and varied CHFR expression in ovarian cancer cell lines. (**A**) formalin fixed, paraffin embedded HeLa cells, which are known to lack CHFR, and HeLa cells stably transfected with plasmid encoding Myc epitope-tagged CHFR, were stained with mAb #10 as described in the Methods. (**B**) aliquots containing 50 µg of total cellular protein from cells shown in A were subjected to SDS-polyacrylamide gel electrophoresis, transferred to nitrocellulose, and probed with CHFR mAb #10 or, as a loading control, rabbit polyclonal anti-RAF1 antibody. For original blots see Figure S3. (**C**), Whole cell lysates from various ovarian cancer cell lines (50 µg of total protein) were subjected to SDS-PAGE followed by immunoblotting with antibodies that recognize the indicated antigens. Numbers at the left indicate molecular weight markers in kDa. For original blots see Figure S3. (**D**) untreated ovarian cancer cell lines were formalin fixed, paraffin embedded and stained with mAb #10.

Based on previous studies showing an association between CHFR protein levels and taxane resistance in various cancers both in vitro [16,21,27,37] and in the clinical setting [26], we examined the relationship between CHFR expression and clinical outcome in patients with epithelial ovarian cancer. Although the latter study used a commercial anti-CHFR antibody, that reagent was not credentialed for immunohistochemistry (IHC) by the supplier and is no longer available. Accordingly, to provide a suitable reagent for CHFR IHC, we raised a murine monoclonal anti-CHFR antibody (termed clone #10) against purified, recombinant CHFR that was treated with formaldehyde. Consistent with previous descriptions of CHFR expression [16], this antibody failed to react with parental HeLa cells but reacted with CHFR-transfected cells to yield strong nuclear and somewhat weaker cytoplasmic staining (Figure 1A). On immunoblots, this antibody recognized the 95 kDa CHFR protein [16] but not any other proteins in these cells (Figure 1B). In further studies, whole cell lysates from a series of ovarian cancer cell lines were subjected to immunoblotting for CHFR and some of its substrates, including Aurora A and KIF22 [10,13]. This analysis indicated that CHFR levels varied extensively among ovarian cancer cell lines, with Ovcar5 and A2780 cells showing particularly low levels (Figure 1C, lanes 2 and 4). Staining (performed for all lines but shown for Ovcar5 and A2780) again paralleled the protein levels (Figure 1D and Figure S1). As was the case with HeLa cells, introduction of CHFR cDNA into A2780 cells restored staining (Figure S1). These untransfected and CHFR-transfected A2780 cells served as negative and positive controls, respectively, in subsequent IHC staining. 

### 2.2. CHFR Expression Varies in Clinical Ovarian Cancer

#### 2.2.1. Clinical Characteristics

CHFR staining was evaluated in surgical resection specimens from 417 previously untreated ovarian cancer patients (Figure S2). Nine hundred and sixty-one cores were scored; 82 patients had one scorable core, 126 patients had two scorable cores and 209 patients had three scorable cores.

Clinical characteristics of the patients included in this analysis are described in Table 1. The majority (307 or 74%) of the patients had high grade serous ovarian carcinomas (HGSOCs). The remaining 110 patients had endometrioid, clear cell or other histologies. Of the 417 patients, 322 were treated at Mayo Clinic with primary debulking surgery followed by platinum/taxane adjuvant therapy using standard regimens (see Methods). This cohort included 249 patients with HGSOC, 64 with high grade non-serous ovarian cancers, and nine with low grade ovarian cancers (Figure S2).

#### 2.2.2. Relationship of CHFR Expression to Tumor Histology, Grade and Debulking Status

**Figure 2:**
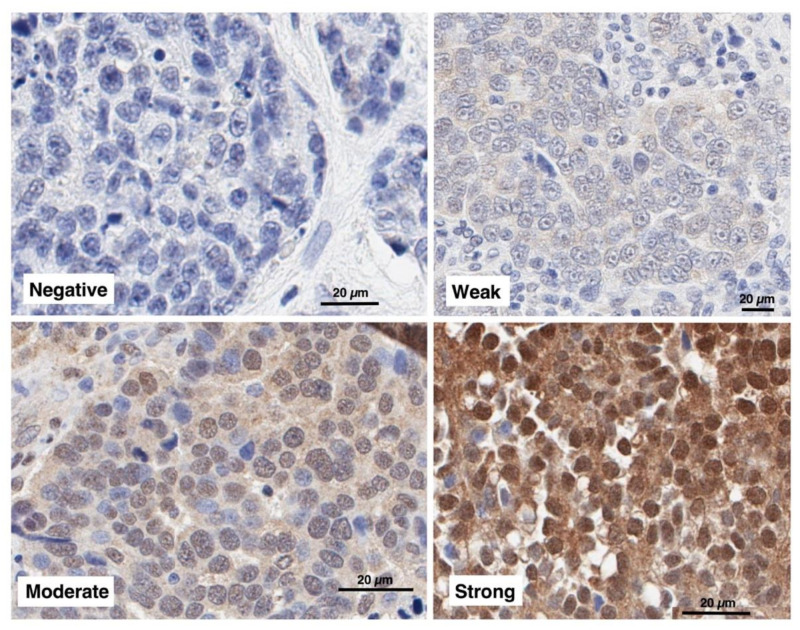
CHFR expression in clinical ovarian cancer. Surgical resection samples from cases of previously untreated epithelial ovarian cancer were stained with the antibody characterized in Figure 1. Examples of negative, weak, moderate and strong staining are shown.

As seen in Figure 2, expression of CHFR varied among different ovarian cancer specimens. To provide a quantitative measure of this variation, the percentages of tumor nuclei in each sample that stained negative, weak, moderate or strong were estimated; and a modified H score was calculated. Modified H scores for the 417 ovarian cancers were then compared among different groups of tumors.

As indicated in Table 2, HGSOCs had a higher H score (median = 220, IQR: 180–260) compared to non-high grade serous histologies (median = 193, IQR: 160–244; *p* = 0.0048). Early-stage tumors (stages 1 and 2) had lower CHFR expression (median = 200, IQR: 150–244) than more advanced cancers (stages 3 and 4) (median = 220, IQR: 180–260; *p* = 0.016). Low grade tumors (grade 1) (median = 160, IQR: 110–180) also had a lower H score than the high grade tumors (grades 2 and 3) (median = 220, IQR: 180–260; *p* = 1.4 × 10^−5^). There was no difference in H scores based on optimal (median = 215, IQR: 170–260) vs. sub-optimal debulking status (median = 210, IQR: 160–250; *p* = 0.487).

#### 2.2.3. CHFR Expression, Overall Survival and Time to Progression in High Grade Serous Ovarian Cancer

**Figure 3:**
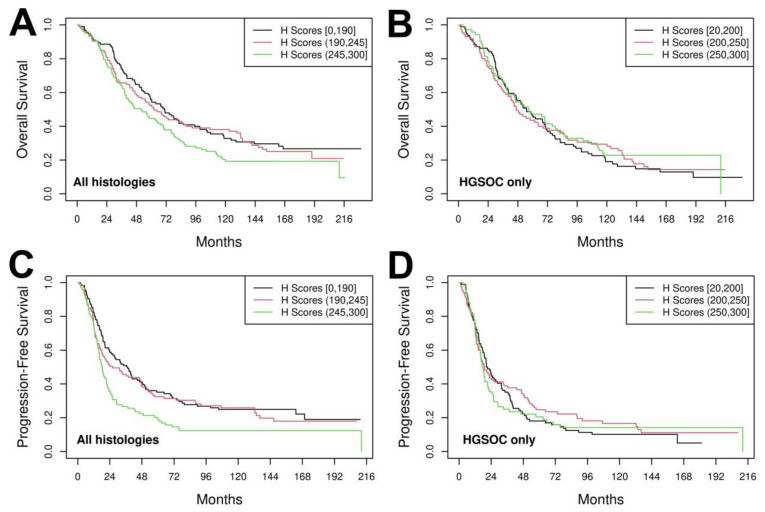
CHFR expression, overall survival, and time to progression after surgery followed by platinum/taxane therapy. Kaplan–Meier curves showing the relationship between CHFR expression categorized by tertiles and overall survival (**A**,**B**) or time to progression (**C**,**D**) in all women with high grade ovarian cancer (**A**,**C**) or those with HGSOC (**B**,**D**).

Further analysis of the relationship between CHFR and treatment response focused on the 322 patients treated with primary debulking surgery followed by platinum/taxane therapy at our institution (Figure S2). Cox proportional hazard regression was utilized to evaluate the relationship between CHFR expression and OS. In this population, the association between CHFR expression and OS was not statistically significant regardless of whether we adjusted for stage and debulking status [hazard ratio for increase in CHFR from 25th to 75th percentile (HR) 1.13, *p* = 0.17 unadjusted; HR = 1.07, *p* = 0.44 adjusted]. Unadjusted Kaplan Meier curves show the distribution of OS by CHFR expression tertiles (Figure 3A). Likewise, after adjustment for stage and debulking, there was also no significant association (HR = 0.96, *p* = 0.62) between CHFR expression and OS (Figure 3B) in the subset of 249 patients with HGSOC who received surgery followed by platinum/taxane adjuvant therapy at Mayo Clinic. 

Because OS reflects the integrated effects of all therapies and not just initial response to platinum/taxane adjuvant therapy, we also examined the relationship between CHFR expression and time to progression (TTP). Platinum/taxane-treated patients with low cancer cell CHFR expression tended to have a longer TTP compared to those with higher CHFR expression (HR = 1.18, *p* = 0.056; Figure 3C). This was no longer significant after adjusting for stage and debulking status (HR = 1.11, *p* = 0.20). Further analysis in HGSOC, the only histological subgroup large enough for meaningful analysis, showed that there was no significant association between CHFR expression and TTP (unadjusted HR = 1.01, *p* = 0.91; adjusted HR = 1.01, *p* = 0.91, Figure 3D). 

#### 2.2.4. CHFR Expression and Response of Ovarian Cancer PDXs to Paclitaxel Monotherapy

**Figure 4:**
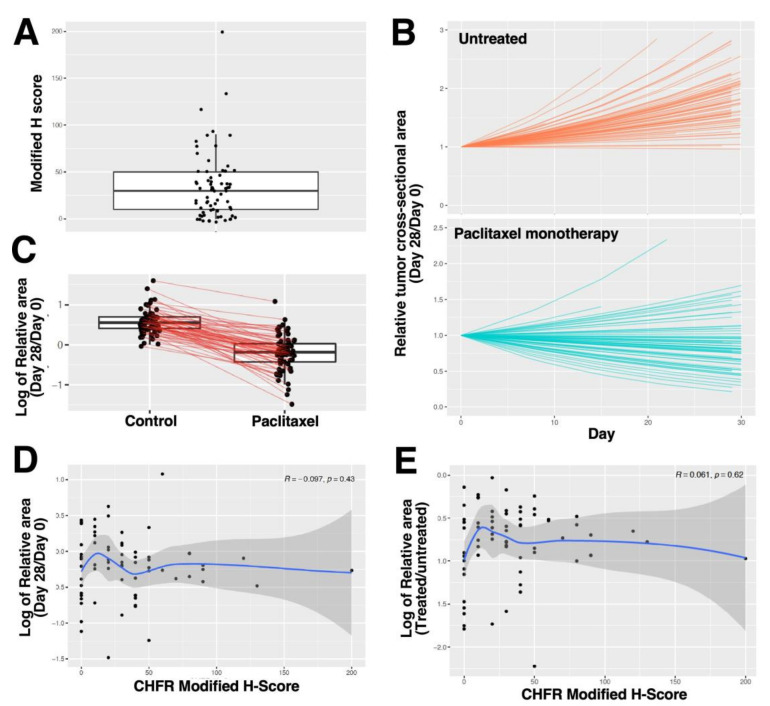
Relationship between CHFR H-score and response of ovarian cancer PDXs to second-line paclitaxel. (**A**) distribution of H scores in pre-treatment ovarian cancer PDXs. (**B**) growth kinetics of 69 individual PDXs models as assessed by transabdominal ultrasound. For each PDX model, 5–8 mice were treated with paclitaxel monotherapy. Model estimated mean maximum cross-sectional area was determined at each time point and divided by the maximum cross-sectional area of tumor in the same PDX model immediately before treatment (Day 0) and plotted as a function of time to generate each curve. Top panel, untreated controls. Bottom panel, the same models treated with 15 mg/kg paclitaxel weekly. (**C**) model estimated mean cross-sectional area on Day 28 relative to Day 0 for each of 69 models that were untreated (left) or treated with paclitaxel (right) on a log_2_ scale; red lines connect paired datapoints. Thus 0 indicates unchanged mean area, 1 indicates a 2-fold increase in mean cross sectional area, and -1 indicates a 2-fold decrease in mean cross-sectional area. (**D**) ratio of model estimated mean cross-sectional area on Day 28 relative to Day 0 in mice treated with paclitaxel, plotted on a log_2_ scale, as a function of mean CHFR H-score for each of 69 separate PDX models. Spearman R together with loess smoother and 95% confidence interval are shown. (**E**) mean Day 28 cross-sectional areas of the paclitaxel-treated mice compared to the mean Day 28 cross-sectional areas of the diluent-treated mice of the same PDX model plotted on a log_2_ scale, as a function of mean CHFR H-score for each of 69 separate PDX models. Spearman R together with loess smoother and 95% confidence interval are shown.

In view of the strong preclinical data associating CHFR with paclitaxel sensitivity (see Introduction), as well as the recent report implicating a UBC13-DNMT1-CHFR pathway in paclitaxel sensitivity of clinical ovarian cancer [35], we were concerned that inclusion of a platinum compound in the treatment might be confounding an underlying relationship between paclitaxel sensitivity and HGSOC response. Because the vast majority of patients receiving paclitaxel (alone or in combination with bevacizumab) for relapsed ovarian cancer do not have pretreatment biopsies immediately prior to initiation of paclitaxel therapy, we turned to a unique set of PDXs treated with paclitaxel monotherapy (Table 1) to further assess the relationship between CHFR expression and response. In these intraperitoneal cancer models, tumor area was measured by transabdominal ultrasound during the course of weekly paclitaxel monotherapy. Analysis of specimens from 69 individual PDX models, of which 60 (87%) were HGSOC, revealed that CHFR H-scores in pretreatment specimens varied from 0 to 200 (Figure 4A). The PDXs also displayed a range of responses (Figure 4B). In particular, untreated PDXs had a mean cross-sectional area on Day 28 that ranged from 0.96 to 4.93 times the mean cross-sectional area of the same models on Day 0 (the day treatment started), whereas paclitaxel treated PDXs had a mean cross-sectional area on Day 28 that ranged from 0.23 to 2.94 times the mean cross-sectional area on Day 0. In other words, all tumors slowed their growth, but only a subset of the models showed evidence of regression on therapy (Figure 4C). Importantly, there was no association between CHFR expression and response to paclitaxel monotherapy as measured by relative tumor cross-sectional area 4 weeks after initiation of therapy relative to the same tumors prior to treatment (Figure 4D) or relative to untreated tumors at the same timepoint (Figure 4E).

## 3. Discussion

As described in the Introduction, the *CHFR* locus has been shown to be widely methylated in a variety of cancers, including ovarian cancer [34]. To the extent that hypermethylation results in diminished gene expression, cells with lower CHFR would be expected to be more sensitive to taxanes [16,21]. Further studies have shown that *CHFR* methylation is mediated by DNMT1 [35,38], which in turn is protected from proteasome-mediated degradation by UBC13 [35]. Importantly, UBC13 expression has been reported to correlate with survival of ovarian cancer patients after paclitaxel-containing therapy, leading to the concept that a UBC13-DNMT1-CHFR pathway modulates paclitaxel sensitivity [35] and providing further impetus for assessing the relationship between CHFR and ovarian cancer outcome in the present study. 

In order to perform IHC staining, we developed a new monoclonal anti-CHFR antibody. This reagent showed a very low signal in FFPE tissue culture cells with low endogenous CHFR expression and a robust signal in FFPE cells with high CHFR levels due to either endogenous expression or transfection with CHFR cDNA (Figure 1 and Figure S1). Each subsequent staining run contained paired A2780 samples with low and high CHFR expression (Figure S1).

When CHFR expression was examined in ovarian carcinomas, a range of expression was observed (Figure 2, Table 2). There was a strong association between CHFR and tumor stage (Table 2). However, once OS data were adjusted for tumor stage and debulking status, there was no significant association between CHFR staining and OS in either the overall population or the HGSOCs, the most common ovarian cancer subtype. 

Because these results were unanticipated, we also examined the association between CHFR expression and paclitaxel response in 69 distinct ovarian cancer PDX models, 60 of which were derived from HGSOCs. Results of this analysis likewise failed to find any relationship between CHFR expression and paclitaxel response (Figure 4). Based on these results, it appears unlikely that CHFR expression will be a useful marker of paclitaxel response in HGSOC.

These results appear to be at odds with the conclusions of Zhang et al. [35], who indicated that high levels of UBC13 protein, acting through DNMT1 to suppress CHFR expression, are associated with improved response of ovarian cancer to paclitaxel-containing therapy. In contrast to their report, the present study directly examined the expression of CHFR rather than a CHFR regulatory protein, specified the “paclitaxel-containing therapy” patients received (see Methods), and performed subgroup analysis that focused on HGSOC, the most common subtype of ovarian cancer treated with paclitaxel. Our results indicated that CHFR is higher in the highest risk histology (HGSOC) and in ovarian cancer that is high grade and high stage (Table 2). Once well-known adverse prognostic factors such as stage and debulking status were taken into account, the relationship between CHFR expression and OS or TTP was not significant. To evaluate the possibility that inclusion of cisplatin or carboplatin in the chemotherapy had confounded an underlying relationship between CHFR expression and treatment outcome, we also conducted a large PDX study examining the relationship between CHFR expression and response to second-line paclitaxel monotherapy (the setting where paclitaxel is used as a single agent in ovarian cancer). These efforts likewise failed to identify any independent role for CHFR as a biomarker of paclitaxel resistance (Figure 4). 

One of the limitations of the present study is the relatively small number of high grade non-serous ovarian cancer cases studied. The mixture of histologies reflects the occurrence of subtypes in our ovarian cancer practice. While the number of HGSOC cases studied was sufficient to rule out an important correlation between CHFR protein expression and TTP or OS after correction for stage and debulking status, we cannot rule out an association between CHFR expression and TTP in individual non-serous histologies because of limited size of the cohorts.

It is also important to emphasize that the lack of correlation between CHFR protein expression and response shown in the present study might be limited to ovarian cancer. The previously reported association between *CHFR* promoter methylation and response to taxanes in lung, gastric and colorectal cancer certainly raises the possibility that CHFR protein levels might predict taxane sensitivity in other tumor types. Further studies are required to assess this possibility. Needless to say, the new antibody for IHC produced in the present study can certainly facilitate these further investigations.

In summary, we have generated and credentialed an antibody that permits detection of the CHFR protein by IHC in clinical material. When this reagent was applied to clinical ovarian cancer specimens, we observed a strong correlation between CHFR expression and HGSOC histology as well as stage. After correction for stage and debulking status, we found no correlation between CHFR levels and response of HGSOC to therapy as assessed by TTP or OS. This finding was corroborated in a separate study in which ovarian cancer PDXs were treated with paclitaxel monotherapy.

## 4. Methods

### 4.1. Reagents and Antibodies

Paclitaxel and geneticin were from Millipore-Sigma (St. Louis, MO, USA). All other reagents were obtained as described previously [39]. Antibodies to PLK1, KIF22, RAD51 and RAF1 were purchased from BD Biosciences (San Jose, CA, USA), Abcam (Cambridge, MA, USA), ThermoFisher (Rockford, IL, USA) and Santa Cruz Biotechnology (Dallas, TX, USA), respectively. 

Murine monoclonal antibodies to CHFR and Aurora A [10,40] were raised using previously described strategies [41,42]. To generate the anti-CHFR antibody, the CHFR open reading frame was cloned into pGEX-4T-1. Recombinant GST-CHFR was induced and affinity purified on glutathione-agarose [43], treated with 3.7% formaldehyde in PBS at 4 °C for 30 min and dialyzed against PBS prior to use as an immunogen. Five female BALB/c mice were immunized by subcutaneous injection of 50 μg of antigen in complete Freund’s adjuvant followed by subcutaneous antigen applications in incomplete Freund’s adjuvant biweekly. After the fourth immunization, a mouse was boosted by intravenous injection of 50 μg of antigen into the tail vein. Three days later 10^8^ spleen cells were fused with FOXNY myeloma cells using PEG1500 (Roche Molecular Biochemicals, Mannheim, Germany). Cells were resuspended in hypoxanthine-aminopterin-thymidine (HAT) selection medium. At day 10 after fusion, hybridoma culture supernatants were screened by immunoperoxidase staining of paired parental (low CHFR) and myc-CHFR-transfected (high CHFR) HeLa cells (kindly provided by Junjie Chen, M.D. Anderson Cancer Center, Houston, TX, USA). For this screening, the paired HeLa lines were formalin fixed, paraffin embedded and sectioned. The anti-CHFR antibody described here is designated clone #10 and is now available from Thermo-Fisher Scientific as catalog #MABS180. 

### 4.2. Tissue Culture

Ovarian cancer cell lines were obtained as follows: Ovcar5 and Ovcar8 from Dominique Scudiero (NCI Frederick, Frederick, MD, USA); PE01 and PE04 from Fergus Couch (Mayo Clinic, Rochester, MN, USA); A2780 and SKOV3 from Viji Shridhar (Mayo Clinic, Rochester, MN, USA) and Ovcar3 from American Type Culture Collection (Manassas, VA). All lines were authenticated by short tandem repeat profiling in the Mayo Clinic Cytogenetics Core. Cells were propagated in the following media: Ovcar5 and Ovcar8 in RPMI 1640 medium containing 10% (*w*/*v*) heat-inactivated fetal bovine serum (FBS), 50 U/mL penicillin G, 50 µg/mL streptomycin and 1 mM glutamine (medium A); Ovcar3 and A2780 in medium A with 10 µg/mL insulin (medium B); and SKOV3 in Dulbecco’s modified essential medium with 10% FBS, 50 U/mL penicillin G, 50 µg/mL streptomycin and 1 mM glutamine (medium C).

For stable transfection, 1 × 10^7^ A2780 cells in antibiotic-free medium B supplemented with 12.5 mM HEPES (pH 7.4) were transfected with plasmid encoding human CHFR fused at its N-terminus with the S peptide and streptavidin binding protein epitopes using a BTX830 electroporator delivering a 10 msec pulse at 320 V. Following plating at 1 × 10^6^ cells per 100 mm tissue culture dish, cells were incubated for 48 h in medium B, then switched to medium B containing 600 µg/mL geneticin for 10 days before individual clones were isolated using cloning rings. Expression of CHFR was verified by immunoblotting as described below. 

### 4.3. Immunoblotting

To prepare whole cell lysates, cells were washed in ice cold serum-free RPMI 1640 medium containing 10 mM HEPES (pH 7.4 at 4°) and dissolved in lysis buffer consisting of 6 M guanidine hydrochloride, 50 mM Tris-HCl (pH 8.5 at 20 °C) 10 mM EDTA, 1 mM PMSF, and 1% (*v*/*v*) 2-mercaptoethanol. After reaction with iodoacetamide, samples were dialyzed sequentially into 4 M urea and 0.1% (*w*/*v*) sodium dodecylsulfate (SDS) [44], lyophilized and resuspended in SDS sample buffer [4 M urea, 2% (*w*/*v*) SDS, 62.5 mM Tris-HCl (pH 6.8)] at 5 mg protein/mL (assayed by the bicinchoninic acid method). Aliquots containing 50 µg of protein were separated by SDS-PAGE, transferred to nitrocellulose and probed with antibodies as described [45].

### 4.4. Clinical Ovarian Cancer Specimens

Patients were prospectively enrolled in the IRB-approved Mayo Clinic Biorepository for Ovarian Cancer Research, which allows for the collection of tumor specimens at the time of surgical debulking as well as ongoing clinical data and outcomes. Triplicate pretreatment tumor samples from women who underwent an initial debulking surgery at Mayo Clinic Rochester between 1999–2009 were arrayed on six TMAs. Formalin-fixed paraffin embedded tissue blocks were cut into 4-µm sections. The relationship between CHFR expression and TTP or OS was analyzed in a subset of 322 women with epithelial ovarian cancers who were treated with primary surgical resection followed by platinum/taxane adjuvant therapy on widely used regimens [46,47].

### 4.5. Treatment of Ovarian Cancer PDXs with Paclitaxel Monotherapy

For all PDX studies, freshly accessioned human ovarian cancer was passaged into female SCID Beige mice (C.B-17/IcrHsd-*Prkdc^scid^Lyst^bg-^J;* Envigo, Indianapolis, IN, USA) as previously described [48]. Briefly, 0.1–0.2 cc of minced tumor was prepared in 1:1 ratio with McCoy’s 5A Modified Medium (Cat# MT-10-050-CV, Corning Life Science) before intraperitoneal injection. Establishment of tumors was assessed by weekly abdominal palpation. Tumor cross-sectional areas were followed by transabdominal ultrasound. 

To see that PDXs were exposed to paclitaxel monotherapy as second-line treatment, the clinical situation in which paclitaxel or nab-paclitaxel is employed as monotherapy, PDXs were first treated with a platinum/taxane doublet and then treated with paclitaxel monotherapy upon regrowth. At an area of 0.3–0.5 cm^2^, mice were randomized to tumor harvest for assessment of baseline characteristics or to treatment with standard of care chemotherapy, which consisted of carboplatin (51 mg/kg) plus paclitaxel (15 mg/kg) given weekly for 2–4 weeks. At the time of regrowth after this standard therapy, tumors were expanded into multiple mice, which were treated with diluent or paclitaxel (15 mg/kg/week) for 4 weeks. During both treatment phases, mice were examined daily to assess activity level, ascites development and ability to access food and water. Mice were weighed weekly and monitored for tumor size by transabdominal ultrasound [48]. At the conclusion of the treatments, mice were euthanized using lethal CO_2_ overdose followed by cervical dislocation, which was consistent with American Veterinary Medical Association guidelines. Tumors treated with diluent at this second generation were again harvested for characterization.

### 4.6. CHFR IHC and Determination of Modified H-Score

IHC staining was performed in the Pathology Research Core (Mayo Clinic, Rochester, MN, USA) using a Leica Bond RX stainer (Leica Biosystems, Buffalo Gove, IL, USA). After antigen retrieval was performed for 20 min using Epitope Retrieval 1 (Citrate; Leica), slides were incubated in Protein Block (Dako, Carpenteria, CA, USA) for 5 min followed by anti-CHFR antibody diluted at 1:100 in Background Reducing Diluent (Dako) for 60 min. 

Antibody binding was detected using the Envision Flex System (Dako). Slides were rinsed between steps with 1X Bond Wash Buffer (Leica), incubated for 5 min in diaminobenzidine from the Envision Flex System, counterstained for 5 min using Schmidt hematoxylin and molecular biology grade water (1:1 mixture) followed by several rinses in 1X Bond wash buffer and distilled water. Slides were then removed from the stainer, rinsed in tap water for 3 min, dehydrated in increasing concentrations of ethanol and cleared in 3 changes of xylene prior to permanent coverslipping in xylene-based medium.

TMAs were scanned on an Aperio ScanScope AT Turbo (Leica Biosystems, Buffalo Grove, IL, USA). Scanned images along with the size markers were grabbed using Aperio eSlide Manager (Leica) and assembled using Canvas X Draw 7.0.2 (Canvas GFX, Inc., Boston, MA, USA).

CHFR staining was scored in a blinded fashion by two individuals with pathology experience for the percentage of cells with each of 4 intensity levels (% negative, % weak, % moderate, % strong). For each sample, a modified H-Score was calculated as (0 × % Negative) + (1 × % Weak) + (2 × % Moderate) + (3 × % Strong). Because of the proposed relationship between high CHFR expression and poor response to taxanes, the maximum H scores from each of the patients were examined for association with histology, stage, grade, OS and TTP.

### 4.7. Statistical Analysis

Jitter plots of the H-scores by TMA were evaluated to identify whether there were any differences in scoring between TMAs. One of the TMAs had significantly weaker staining than the other TMAs despite a similar mix of cases and was excluded from the analysis. Other exclusions included samples with no scorable cancer on the TMA cores and histological exclusions for non-ovarian, borderline, and borderline malignant mixed tumors, leaving 417 subjects for analysis. 

Differences in H score between clinical characteristics (histology, stage, grade, debulking status) were evaluated using the Kruskal–Wallace rank sum test. OS was defined as time from diagnosis to death due to any causes. TTP was defined as the time from diagnosis to progression or recurrence, i.e., appearance of detectable lesions in patients who achieved complete response, or death. Based on the proposed mechanism of action, OS and TTP analyses were performed only in patients who received platinum and taxane based chemotherapy. Cox Proportional Hazard models with Wald tests were used for hypothesis testing, both unadjusted and adjusted for stage and debulking [49]. Hazard ratios were reported for an increase from the 25th to 75th percentile in the CHFR expression distribution for the patient group being analyzed (e.g., non-HGSOG IQR = (160, 244); HGSOC IQR = (180, 260)). Kaplan–Meier curves for OS and TTP were used to visualize outcomes grouped by maximum H score tertiles. 

For analysis of the PDX study, tumor areas represent the largest cross-sectional area detected in each of 5–8 mice per treatment arm by weekly transabdominal ultrasound. For each PDX, tumor growth trajectories were analyzed by repeated measures analysis implemented via linear mixed effects models. Akaike and Bayesian information criteria together with graphics were used to determine the functional form of the mean model (straight on the natural log scale) and covariance structure (spatial power) as previously described [50]. Model-based mean estimates for subsequent correlation analysis and visualization for each PDX were outputted for two measures: (i) the cross-sectional area of each PDX on day 28 relative to day 0, and (ii) the mean Day 28 cross-sectional areas of the paclitaxel-treated mice compared to the mean Day 28 cross-sectional areas in the diluent-treated mice of the same PDX model. Variation of these two response parameters was then assessed as a function of CHFR H-score via Spearman correlation coefficients and loess smoothers with 95% confidence intervals. Analyses were performed via R [51] and SAS software (copyright 2016, SAS Institute Inc., Cary, NC, USA).

## 5. Conclusions

CHFR expression varies widely among ovarian cancers and correlates with stage, grade and serous histology. After correction for stage and grade, CHFR does not correlate with response in HGSOC, the most common and deadliest subtype. Accordingly, CHFR levels are unlikely to be useful as a predictive or prognostic marker in this disease.

## Figures and Tables

**Table 1:** Patient and cancer characteristics.

Pathology and Treatment	TMA Staining Overall (N = 417)	PDX Staining (N = 69)
Histology		
High Grade Serous	307 (73.6%)	60 (87.0%)
Endometrioid	49 (11.8%)	3 (4.3%)
Clear Cell	29 (7.0%)	4 (5.8%)
Other	32 (7.7%)	2 (2.9%)
Stage		
1	66 (15.8%)	2 (2.9%)
2	32 (7.7%)	2 (2.9%)
3	257 (61.6%)	49 (71.0%)
4	62 (14.9%)	16 (23.2%)
Grade		
1	21 (5.0%)	2 (2.9%)
2	35 (8.4%)	3 (4.3%)
3	361 (86.6%)	64 (92.8%)
Debulking Status *		
Optimal	371(89.0%)	64 (92.8%)
Sub-optimal	46 (11.0%)	3 (4.3%)
Unknown	-	2 (2.9%)
Platinum/Taxane regimen		
Yes	322 (77.2%)	-
No	12 (2.9%)	-
Unknown	83 (19.9%)	-

* Optimal debulking was defined as residual macroscopic disease less than 1 cm. Suboptimal debulking was defined as residual disease deposits greater than or equal to 1 cm.

**Table 2:** Association of CHFR expression with histology, grade, stage and debulking status.

Variable	Group	N	Median	IQR	*p*-Value ^1^
Histology	Non-HGS	110	192.5	(160, 244)	0.0048
	HGS	307	220	(180, 260)	
Stage	1	66	200	(160, 240)	0.053
	2	32	187.5	(137.5, 250)	
	3	257	220	(180, 260)	
	4	62	210	(170, 249)	
Stage Grouped	Early (1 & 2)	98	200	(150, 244)	0.016
	Advanced (3 & 4)	319	220	(180, 260)	
Grade	1	21	160	(110, 180)	5.4 × 10^−5^
	2	35	210	(175, 250)	
	3	361	220	(180, 260)	
Grade Grouped	Low (1)	21	160	(110, 180)	1.4 × 10^−5^
	High (2 & 3)	396	220	(180, 260)	
Debulking Status Grouped	Optimal	371	215	(170, 260)	0.49
	Sub-optimal	46	210	(160, 250)	

^1^ Kruskal-Wallis rank sum test.

## Data Availability

The data presented in this study are available in this article and supplementary material.

## Supplementary Materials

The following are available online at https://www.mdpi.com/article/10.3390/cancers13236043/s1, Figure S1: Characterization of A2780 clones used for IHC staining controls. Figure S2: Ovarian cancers analyzed for the present study. Figure S3: Original blots.
