# Supplementary material for: CHFR and Paclitaxel Sensitivity of Ovarian Cancer
Source: Cancers (Basel). 2021 Nov 30;13(23):6043. doi: 10.3390/cancers13236043 (PMC8657201; doi:10.3390/cancers13236043)
Supplement: Supplementary file 1 [file cancers-13-06043-s001.zip › cancers-1428071-supplementary.pdf]

Supplementary material for

CHFR and Paclitaxel Sensitivity of Ovarian Cancer

Andrea E. Wahner Hendrickson, Daniel W. Visscher, Xiaonan Hou, Krista M. Goergen, Hunter J. Atkinson, Thomas G. Beito, Vivian Negron, Wilma L. Lingle, Amy K. Bruzek, Rachel M. Hurley, Jill M. Wagner, Karen S. Flatten, Kevin L Peterson, Paula A. Schneider, Melissa C. Larson, Matthew J. Maurer, Kimberly R. Kalli, Ann L. Oberg, S. John Weroha and Scott H. Kaufmann

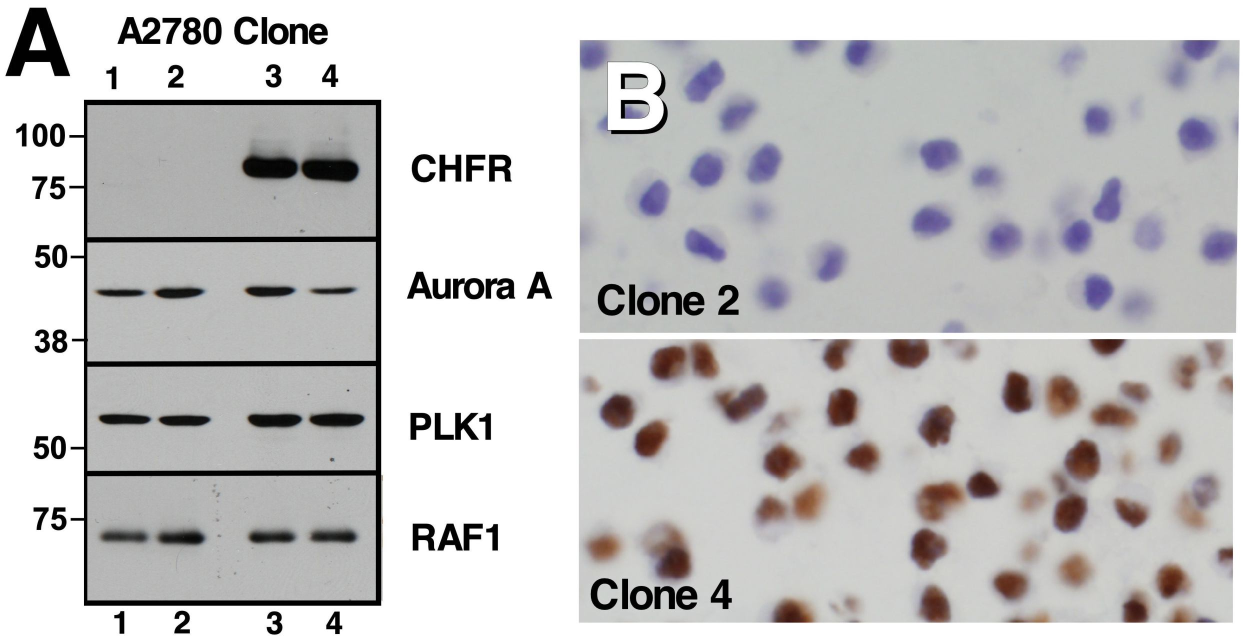

Figure S1. Characterization of A2780 clones used for IHC staining controls. A, A2780 cells were stably transfected with a plasmid containing the CHFR open reading frame behind the constitutively active CMV promoter. Lysates from the resulting clones underwent immunoblotting with the indicated antigens. The housekeeping protein RAF1 served as a loading control. **B,** two of the A2780 clones, one expressing CHFR and one not, were arbitrarily chosen to be formalin fixed, paraffin embedded and stained with anti-CHFR antibody according to the procedure outlined in the Methods. These cells were then included as negative and positive controls in all further IHC staining runs.

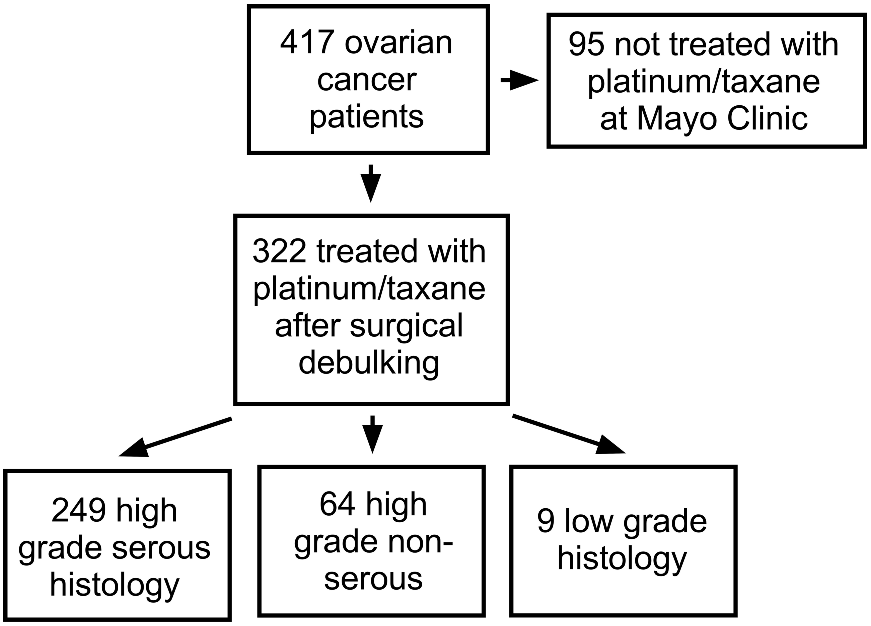

Figure S2. Ovarian cancers analyzed for the present study. The sample set consisted of 417 ovarian cancers, 322 of which were treated with debulking surgery followed by platinum/taxane therapy at Mayo Clinic. The entire set of 417 ovarian carcinomas was considered for analysis of association between CHFR expression and histology, stage, grade and debulking status. Because detailed treatment information and survival data were missing for patients who received chemotherapy away from Mayo Clinic, only 322 cases treated at Mayo Clinic were considered for assessing the association between CHFR expression and time to progression or overall survival.

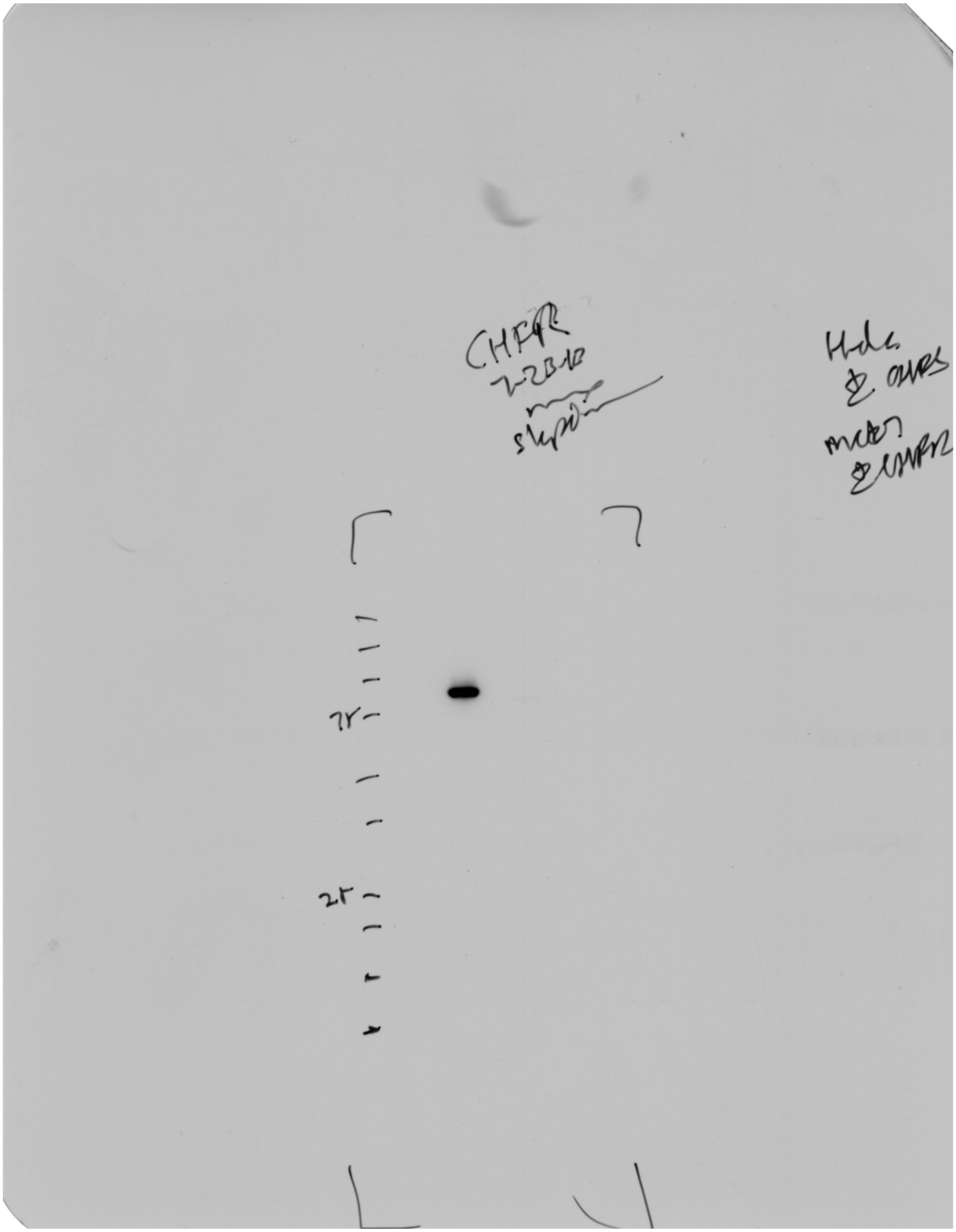

Figure 1B, top panel

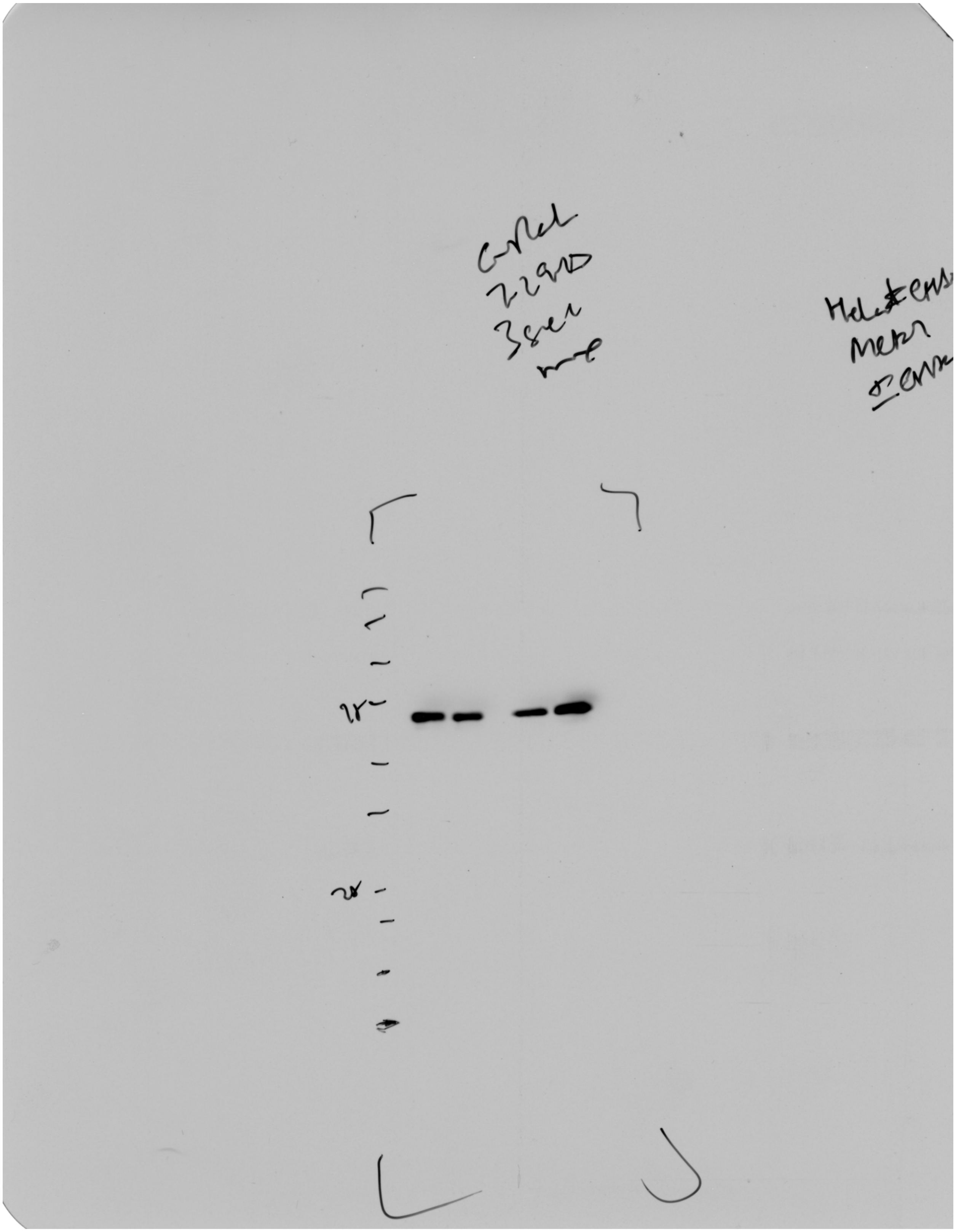

Figure 1B, bottom panel

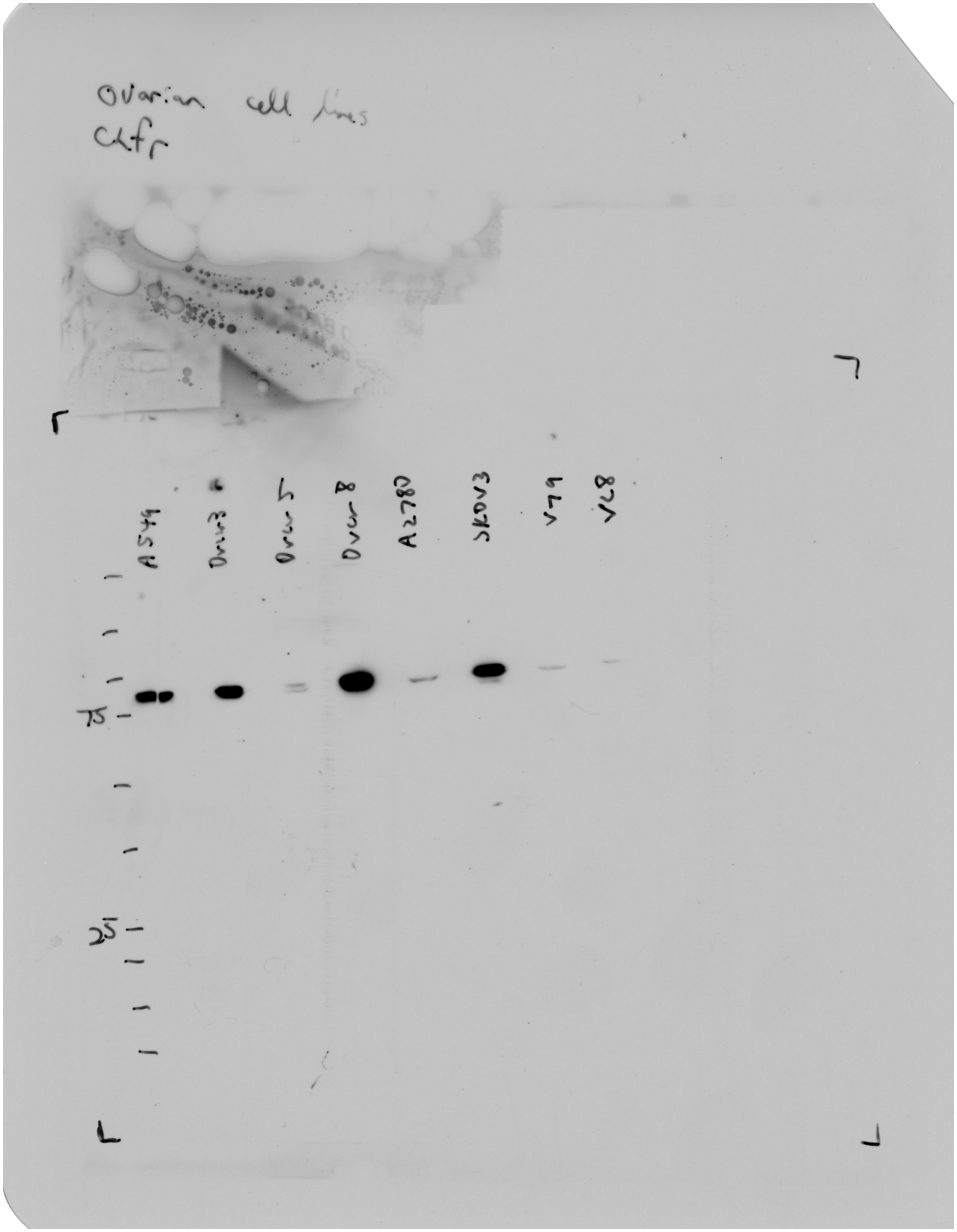

Figure 1C, top panel

Order of lanes in this and subsequent blots: A549 (lung), Ovcar 3, Ovcar 5, Ovcar 8, A2780, SKOV3 followed by four Chinese hamster lines (V79, VC8, AA8, EM9) that react with some antibodies to human proteins but not others.

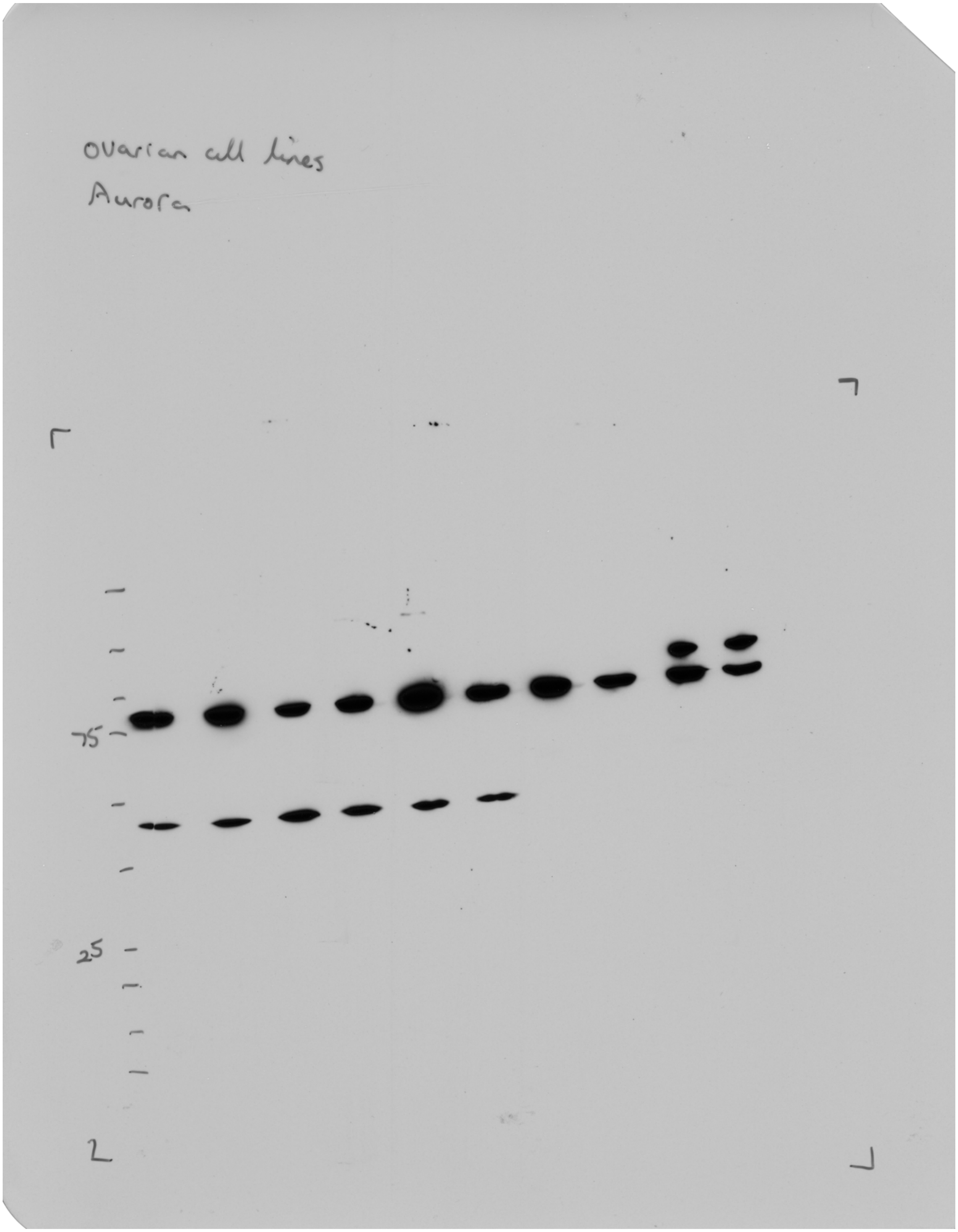

Figure 1C, second panel

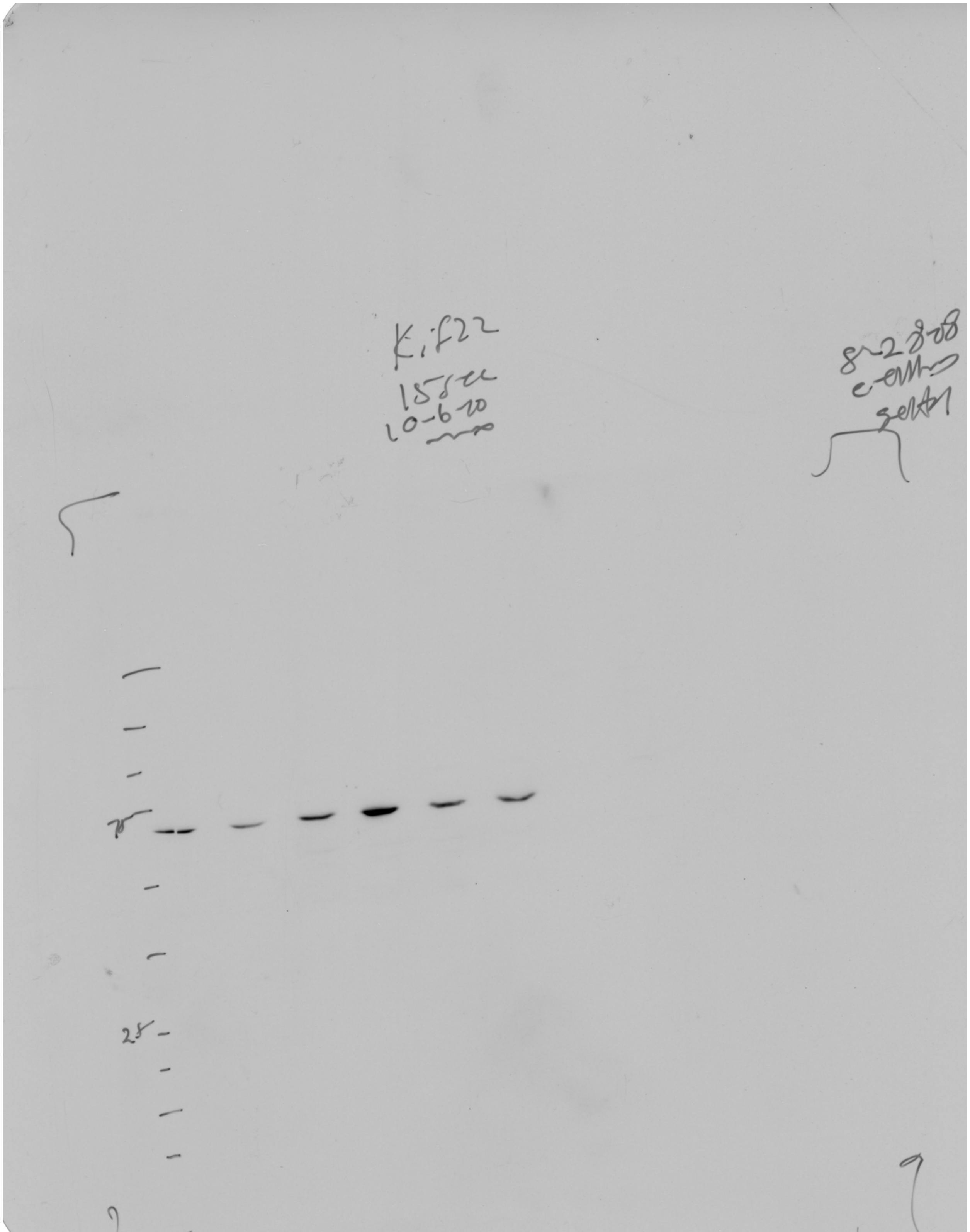

Figure 1C, third panel

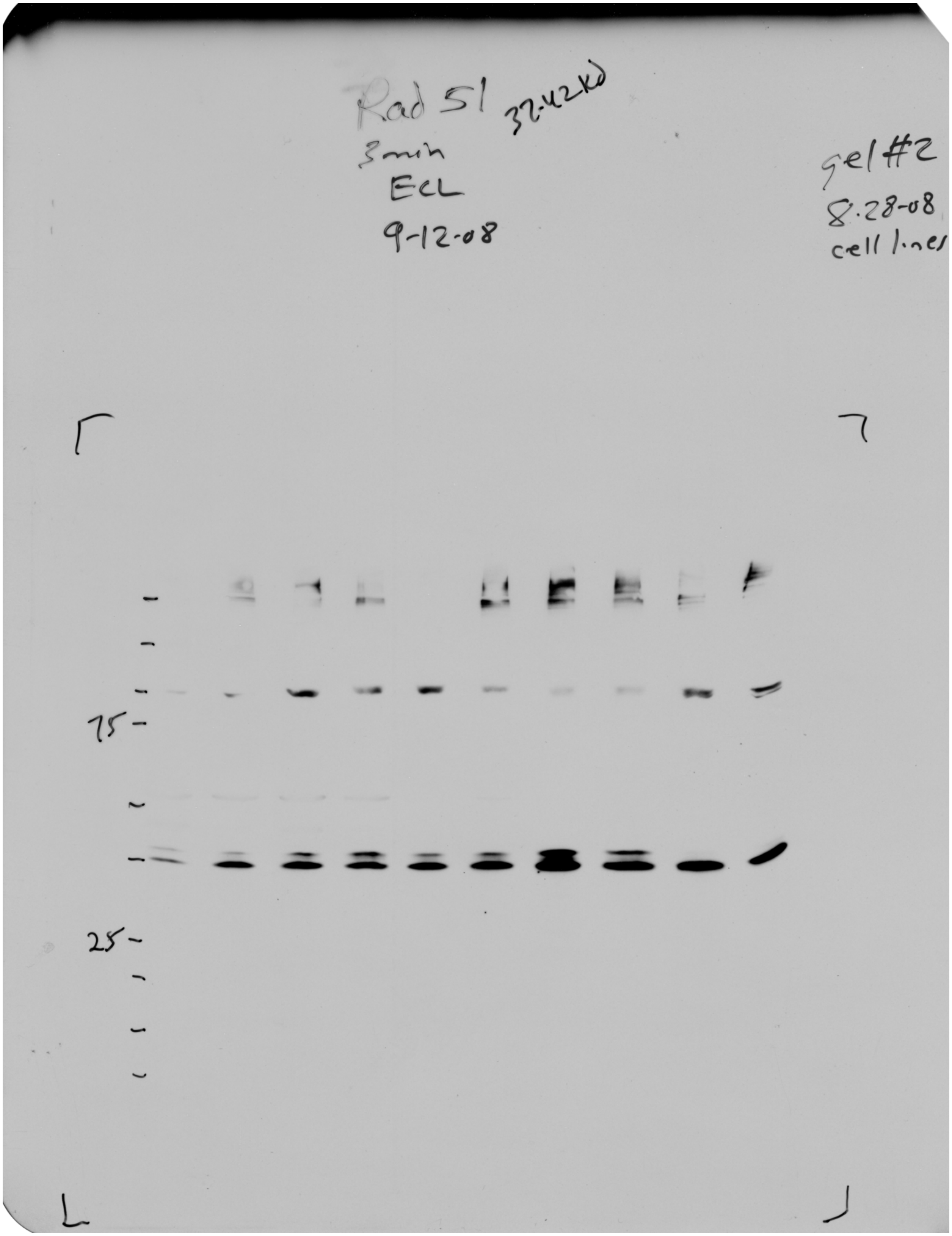

Figure 1C, fourth panel

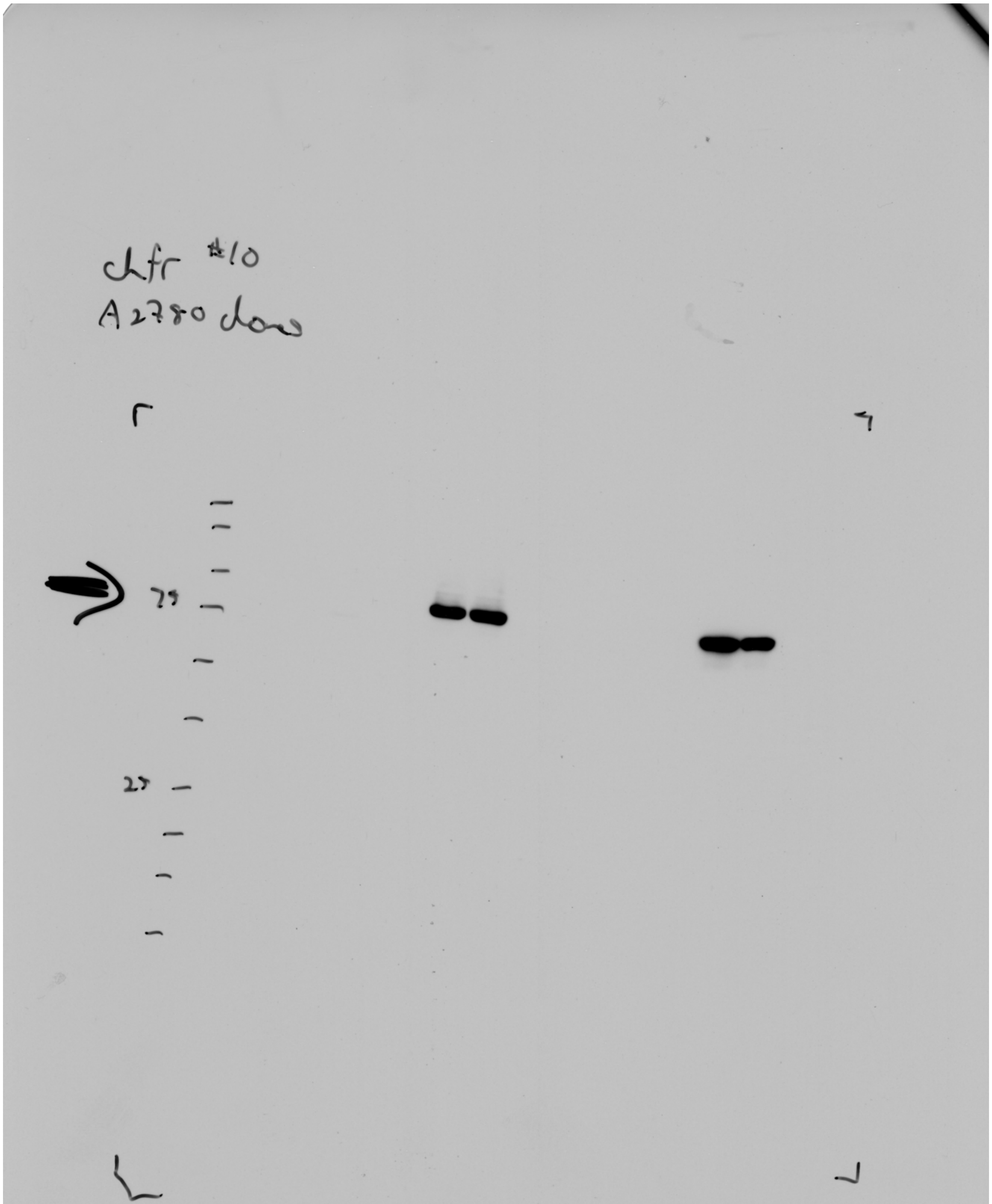

Figure S1A, top panel

Order of samples in this and subsequent panels: Parental A2780, space, two clones with empty vector, space, two clones with S/S CHFR. This is repeated a second time with lysates prepared again on a separate day.

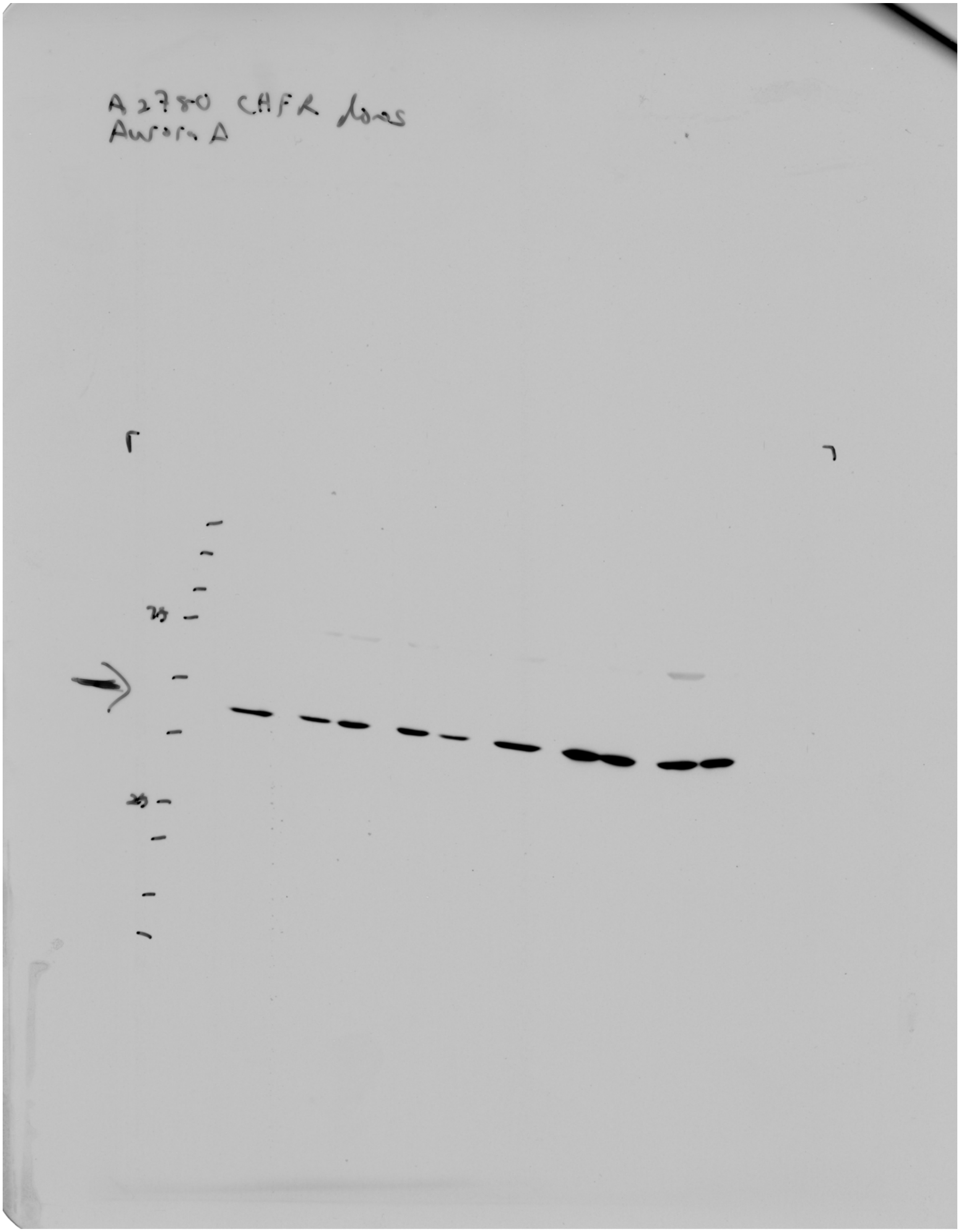

Figure S1A, second panel

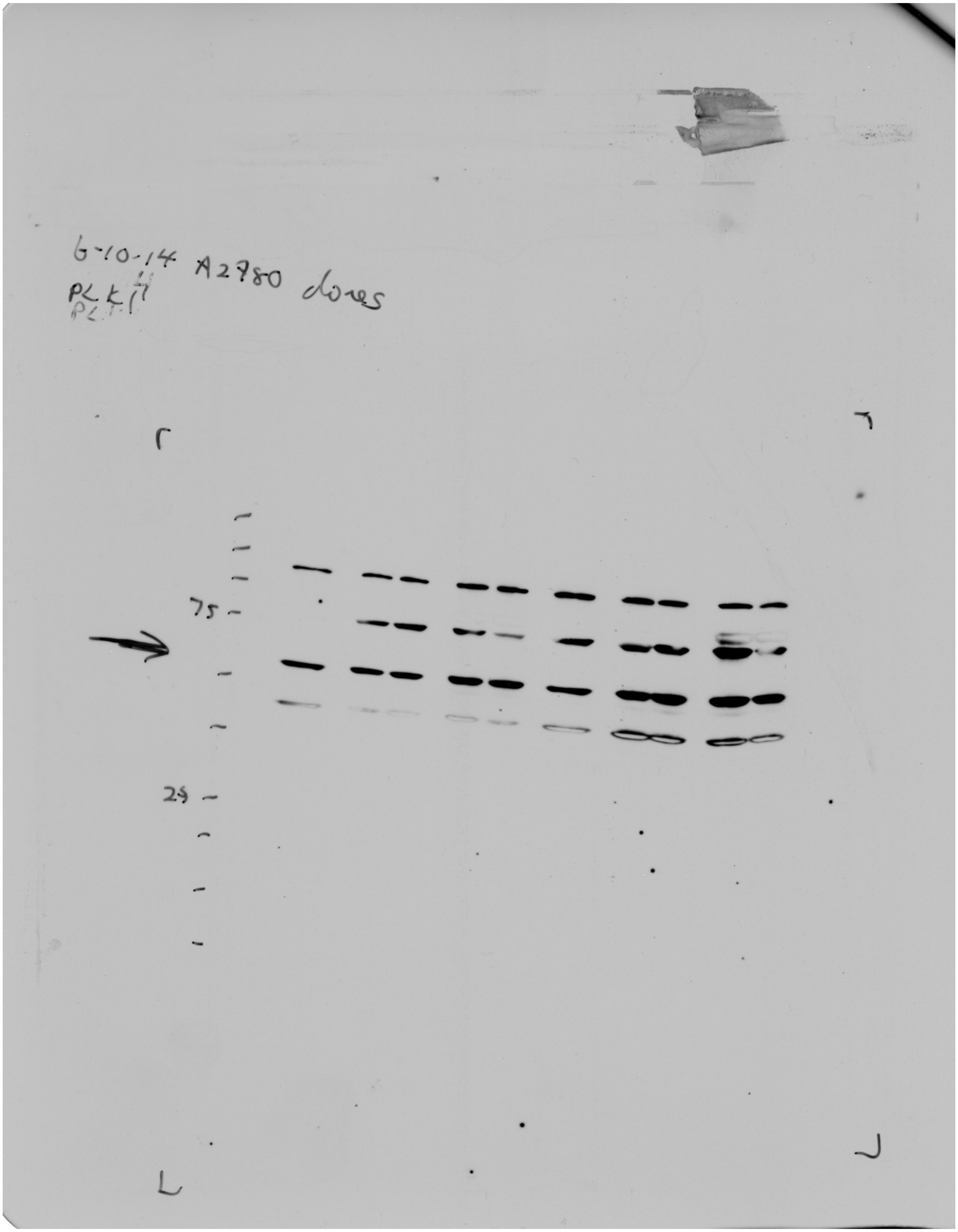

Figure S1, third panel

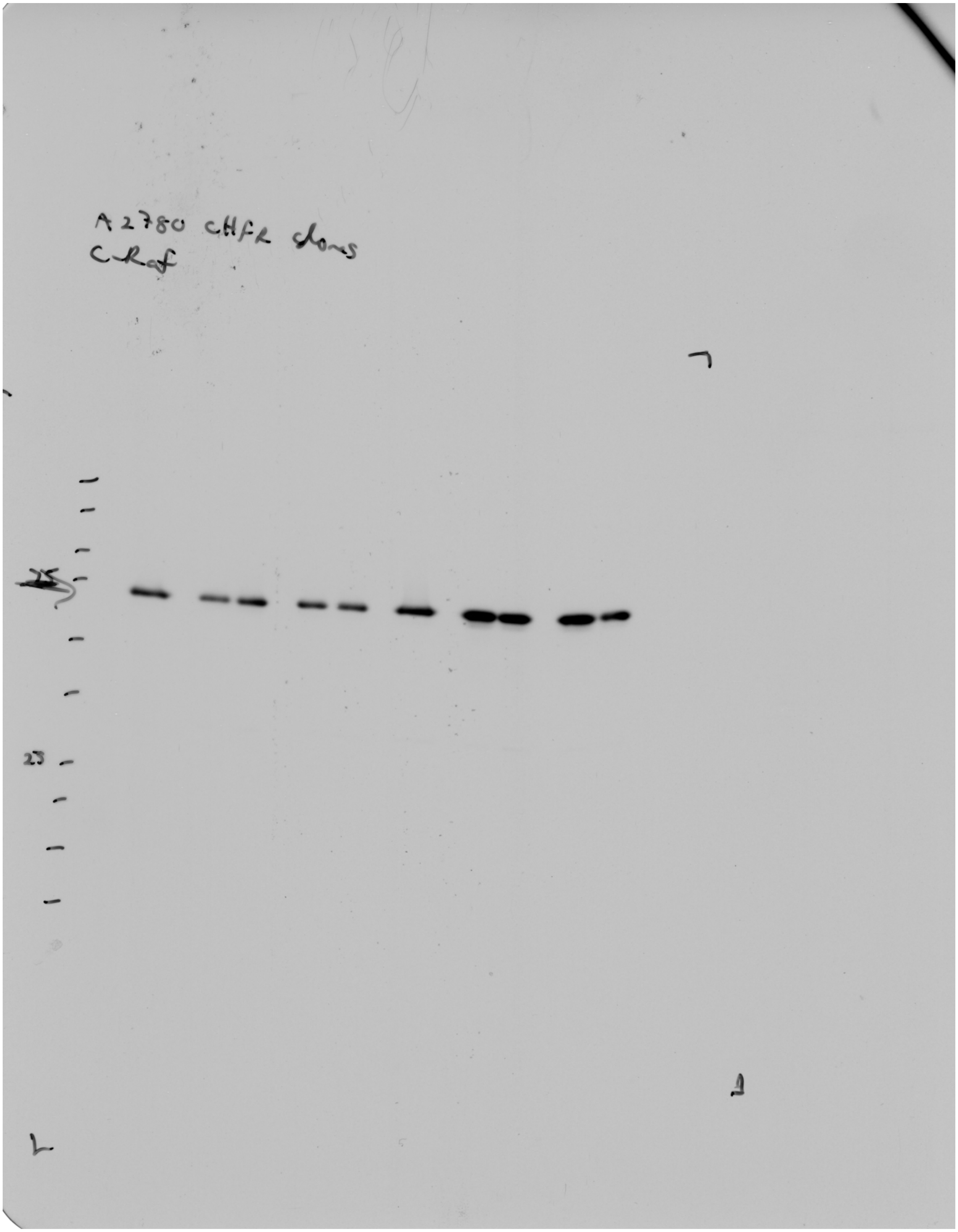

Figure S1A, bottom panel
